# Weird genotypes? Don't discard them, transmissible cancer could be an explanation

**DOI:** 10.1111/eva.12439

**Published:** 2016-12-16

**Authors:** Florentine Riquet, Alexis Simon, Nicolas Bierne

**Affiliations:** ^1^ Université de MontpellierSèteFrance; ^2^ Institut des Sciences de l'EvolutionCNRS‐UM‐IRDMontpellierFrance

**Keywords:** heteroplasmy, infectious cancer, *Mytilus*

## Abstract

Genetic chimerism is rarely considered in the analysis of population genetics data, because assumed to be an exceptionally rare, mostly benign, developmental accident. An unappreciated source of chimerism is transmissible cancer, when malignant cells have become independent parasites and can infect other individuals. Parasitic cancers were thought to be rare exceptions, only reported in dogs (Murgia et al., *Cell*, 2006, *126*, 477; Rebbeck et al., *Evolution*, 2009, *63*, 2340), Tasmanian devils (Pearse and Swift, *Nature*, 2006, *439*, 549; Pye et al., *Proceedings of the National Academy of Sciences*, 2016, *113*, 374), and soft‐shell clams (Metzger et al., *Cell*, 2015, *161*, 255). However, the recent simultaneous report of four new contagious leukemias in marine mollusks (Metzger et al., *Nature*, 2016, *534*, 705) might change the rules. By doubling up the number of naturally occurring transmissible cancers, this discovery suggests they may essentially be missed because not sufficiently searched for, especially outside mammals. We encourage population geneticists to keep in mind infectious cancer when interpreting weird genotypes in their molecular data. It would then contribute in the investigation of how widespread contagious cancer could really be in the wild. We provide an example with our own data in *Mytilus* mussels, a commercially important shellfish. We identified genetic chimerism in a few mussels that suggests the possible occurrence at low prevalence in European *M. edulis* populations of a *M. trossulus* contagious cancer related to the one described by Metzger et al. (*Nature*, 2016, *534*, 705) in populations of British Columbia.

## 1 Transmissible cancers might be more widespread than previously considered

A cancerous lineage initiates when a cell enters a selfish life in an individual, growing and dividing uncontrollably, and usually ends with the death of its host. Despite genomic instabilities, the genome sequence of the cancerous lineage is nearly identical to the host genome from which it derives. A few cancers, however, have acquired the ability to infect new hosts. In this case, parasitic cancer cells are genetically distinct from the host they infect. Infectious cancers have been demonstrated in only two mammal species so far, a group for which cancer is much more studied than in others. A sexually transmitted venereal tumor was reported in dogs (Murgia, Pritchard, Kim, Fassati, & Weiss, 2006) while malignant cells of facial tumors were identified to be transmitted by biting in Tasmanian devils (Pearse & Swift, 2006). Two key elements of transmissible cancer emergence are the survival of malignant cells during transmission from host to host and the resistance to immune attacks in the new host (Ujvari, Gatenby, & Thomas, 2016). From a mammal‐centered point of view, with cells unable to survive in the external environment and with a highly effective self‐recognition system, it looks like incredibly difficult conditions to meet, well explaining the rarity of contagious cancers. When these conditions are met, however, multiple emergences could be expected (Ujvari et al., 2016) as found in the Tasmanian devil (Pye et al., 2016).

More recently, the study of transmissible cancers has made considerable progress when it has been investigated in marine invertebrates, in which knowledge about malignant overgrowth is much less developed than in terrestrial mammals. Metzger, Reinisch, Sherry, and Goff (2015) first reported a transmissible leukemia in the soft‐shell clam *Mya arenaria*. This result implied that malignant cells could survive the transit in seawater and managed to escape the more rudimentary self‐recognition system of this bivalve. As these two conditions seem more likely to be reached in the marine environment, could transmissible cancer be more frequent in marine invertebrates? The answer has just arrived last June, with transmissible cancers identified in multiple marine bivalve species (Metzger et al., 2016). In addition to the previously reported case in *M. arenaria*, hallmarks of transmissible cancers have been found in *Mytilus trossulus* mussels, in *Cerastoderma edule* cockles, and in the golden carpet‐shell clam *Polititapes aureus* (Metzger et al., 2016). These marine bivalves are far from genetically depleted as Tasmanian devil and dog breeds were suspected to be. For instance, *M. trossulus* proved to be the second most polymorphic species in a genomewide survey of genetic diversity conducted in 76 animal species (Romiguier et al., 2014). If that was not astounding enough, the infectious cancer found in *P. aureus* clams proved to originate from another species, the pullet shell clam *Venerupis corrugata*. Surprisingly, no signs of cancer were found in the donor species. This makes the first report of a parasitic cancer that jumped between species. As eight contagious cancers have been reported so far, cross‐species contamination could also well be more frequent than thought.

If transmissible cancer is a widespread phenomenon in the sea, why then did we not detect it earlier? Either because it is a new phenomenon stimulated by new infectious or carcinogenic agents, or most probably because it has simply not been sufficiently searched for.

## 2 Lesson for population genetic analysis, reinterpreting weird genotypes

The analysis of DNA markers is central in the demonstration of transmissible cancer. Old suspicions of transmissible tumor in Syrian hamsters (Cooper, Mackay, & Banfield, 1964) lacked the DNA marker validation. In order to demonstrate a transmissible cancer, genetic differences need to be found between cancerous and host tissues. This needs to be investigated despite difficulties, in mammals that usually display low genetic diversity, but also in bivalves for which host and cancerous cells are often mixed up and co‐amplify (see Extended Data fig. 1 in Metzger et al., 2016). In addition, genetic similarities between cancer cell DNA of different individuals attests infection by the same clone and genetic differences allow identifying cancer lineages that must have emerged independently, as found in Tasmanian devil (Pye et al., 2016) and cockles (Metzger et al., 2016).

Population geneticists manipulate molecular markers every day but often extract DNA from a single tissue and rarely conduct histological inspection. What would happen if a population geneticist co‐amplifies infectious cancer cells with host DNA in one or a few individuals of a sample? Prior to Metzger et al. (2016) publication, infectious cancer would unlikely be considered. Two mitochondrial sequences would most likely be interpreted as contamination or heteroplasmy possibly due to paternal leaking. More than two microsatellite or sequence alleles in heterozygous diploids would have inevitably been interpreted as contamination, paralogous amplifications, or other technical artifacts. With biallelic SNP markers, whatever the typing method (PCR‐based, mass spectrometry, or genotyping‐by‐sequencing), the effect could possibly remain undetected, producing either more or less expected highly heterozygous or hybrid genotypes, or a high rate of missing data owing to the imbalance amplification of the two alleles. Finally, bioinformatics pipelines designed to analyze NGS data and call variants do not usually consider genetic chimerism. As for SNP typing, the effect could simply be either a high rate of missing data or to elevate individual heterozygosity, which would likely remain unnoticed without a dedicated analysis of allelic read counts. Now that we became aware of the possibly high prevalence of contagious cancers in the wild, let us keep it in mind when interpreting weird genotypes and develop routine procedures to track genetic chimerism in our dataset.

## 3 Example with our own data in *Mytilus* mussels

**Figure 1:**
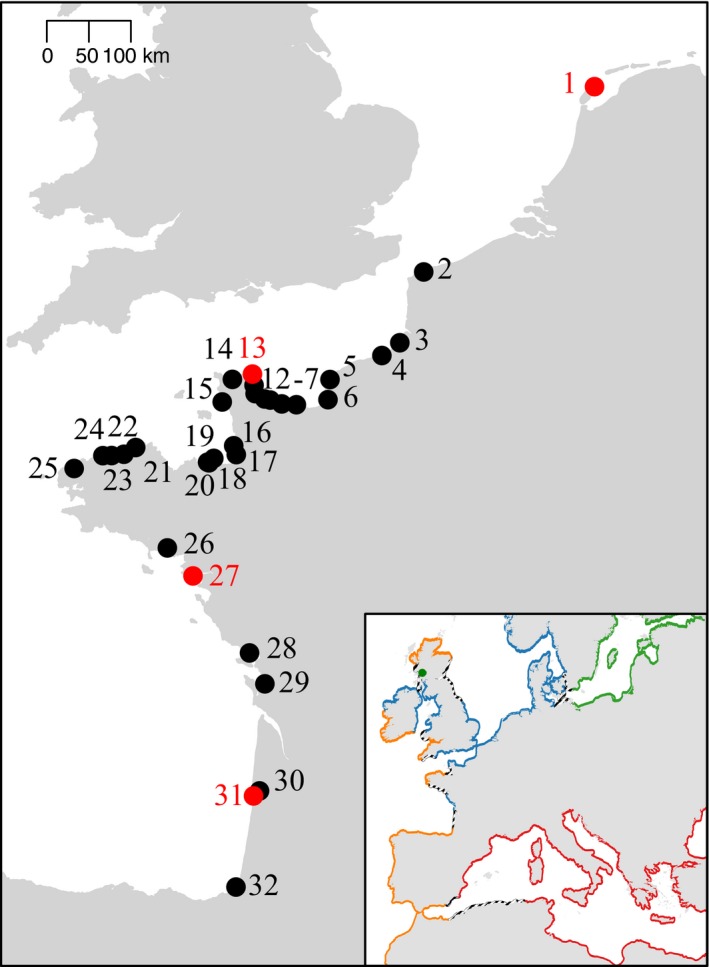
Sampling locations of *Mytillus* mussels, numbered from 1 to 32 following the French Atlantic and Channel coastlines: 1 Wadden Sea, 2 Calais, 3 Ault, 4 Dieppe, 5 Saint‐Jouin‐Bruneval, 6 Villerville, 7 Ouistreham, 8 Le Bouffay, 9 Englesqueville‐la‐Percée, 10 Grandcamp, 11 Ravenoville, 12 Réville, 13 Barfleur, 14 Cherbourg, 15 Carteret, 16 Granville, 17 Sol‐Roc, 18 Rotheneuf, 19 Saint‐Enegat, 20 Dinard, 21 Roc Rouge, 22 Locquémeau, 23 Primel, 24 Roscoff, 25 Guillec, 26 Kerbihan, 27 Pornichet, 28 Aiguillon, 29 Lupin, 30 Arcachon, 31 Banc d'Arguin, and 32 Biarritz. Sites where weird mussel genotypes were observed are displayed with red dots. In the insert, distribution range of *M. edulis* is depicted in blue, Atlantic *M. galloprovincialis* in orange, Mediterranean *M. galloprovincialis* in red, and *M. trossulus* in green, while hybrid zones are represented with black and white stripes

**Figure 2:**
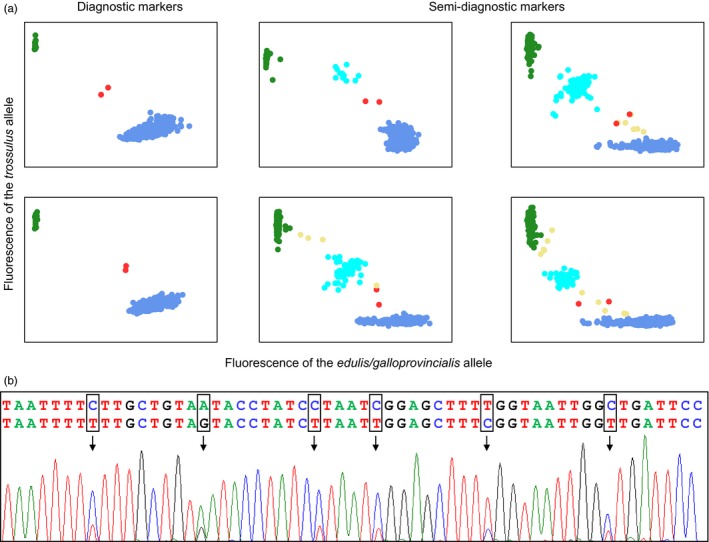
(a) Examples of SNP cluster plots using the KASPar^®^ assay technology for six markers. Green dots: homozygotes for the *trossulus* allele (allele more frequent in *M. trossulus* reference samples), blue dots: homozygotes for the *edulis/galloprovincialis* allele (allele in higher frequency in *M. edulis* and *M. galloprovincialis* reference samples than in *M. trossulus* reference samples), cyan dots: heterozygous genotypes that are not the chimeric mussels, yellow dots: ambiguous genotypes that are not the chimeric mussels, red dots: chimeric mussels. The two markers on the left are diagnostic between *M. trossulus* and *M. edulis/M. galloprovincialis* with one allele fixed in our *M. trossulus* reference samples and another allele fixed in other samples. The four markers on the right are Semi‐diagnostic markers; they are strongly differentiated between reference samples but not differentially fixed such that a few heterozygous individuals are found in parental populations. Chimeric mussels (red dots) systematically deviate from the heterozygote cluster cloud, while other ambiguous genotypes (yellow dots) are different individual for different markers. (b) Chromatogram of a mtCOI sequence showing heteroplasmy. The two sequences corresponding to *M. trossulus* and *M. edulis* alleles are given on top of the trace image. SNPs are framed on the sequences, while black arrows pinpointed SNPs on the trace image

**Figure 3:**
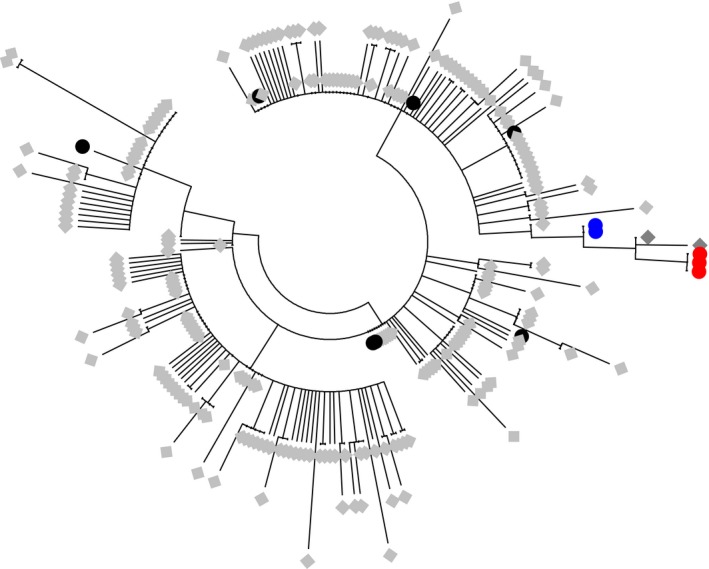
Phylogenic tree realized using MEGA7.0 (Kumar, Stecher, & Tamura, 2016) with all available *M. trossulus* mtCOI sequences plus our two *M. trossulus* mtCOI sequences identified in chimeric mussels. Sequences from chimeric French mussels are depicted with blue circles, neoplastic cell sequences (from Metzger et al., 2016) with red circles, nonneoplastic cell sequences (from Metzger et al., 2016) with black circles, sequences from GenBank with light gray diamonds and sequences from GenBank from Layton et al. (2014) in dark gray diamonds

Metzger et al. (2016)'s publication appeared while we were having difficulties to interpret weird genotypes in our *Mytilus* mussel dataset. Among 938 mussels sampled along the European Atlantic coasts (from the Netherlands to France), we found what we thought to be five hybrid genotypes between *M. edulis* and *M. trossulus*, one in the Wadden Sea, one in Barfleur, one in Pornichet, and the other two in Arcachon (see samples 1, 13, 27 and 31 in the map, Figure 1). However, only *M. edulis* and *M. galloprovincialis* are usually found along these coasts, and the closest *M. trossulus* populations are reported in Scotland and the Baltic Sea (see insert in Figure 1). Contamination was essentially refuted by multiple DNA extractions and amplifications in different laboratories (ours, the ADNid laboratory (http://www.adnid.fr/index.html) to which we subcontracted SNP typing with the Illumina BeadXpress^®^ technology, and the English LGC Genomics laboratory (http://www.lgcgroup.com) to which we subcontracted SNP typing with the KASPar^®^ assay technology). In addition, SNPs were newly developed and had never been amplified anywhere previously. We were therefore considering the possibility of a hidden invasion by *M. trossulus* in unsuspected habitats that we do not usually sample (e.g., deep populations, estuaries, or ports). Note that we did not initially analyze mtDNA as it is not informative to discriminate *M. edulis* and *M. galloprovincialis*. What if these hybrids were in fact chimeric mussels, *M. edulis* individuals contaminated by a transmissible cancer of *trossulus* origin? It could have been a silly idea if not awoken by Metzger et al. (2016)'s scoop. Two “chimeric” mussels have been analyzed with the KASPar^®^ assay technology together with samples from the English Channel and the Wadden Sea, and a reference sample of *M. trossulus* from the Japan Sea. The fluorescence of heterozygous SNPs for these two individuals proved to be consistently biased toward the *edulis* allele when compared to other heterozygous individuals (Figure 2a). Some other individuals sometimes showed deviated fluorescence but never consistently on every marker. A similar tendency was observed for the other three chimeric mussels analyzed with the Illumina BeadXpress technology although the effect was less clear because the fluorescence variance of true heterozygotes was much stronger (data not shown). We therefore sequenced the mtCOI gene following Metzger et al. (2016)'s protocol and analysis. Chromatograms inspection revealed that a *M. trossulus* mtCOI sequence was co‐amplifying with the *M. edulis* sequence in two of the five individuals, although at a lower rate (Figure 2b). This result is a strong argument against the hypothesis of hybridization. The two *M. trossulus* mtCOI sequences were identical and closely related to the Pacific *M. trossulus* parasitic cancer lineage reported in Metzger et al. (2016). A phylogenic tree with all available *M. trossuslus* mtCOI sequences is shown in Figure 3. Two mtCOI sequences from the DNA barcode study of Layton, Martel, and Hebert (2014), and identified as *M. trossulus*, clustered with both ours and Metzger et al. (2016) leukemia cell sequences. Kara Layton kindly sent us the chromatograms of these two sequences, amplified from muscle tissues (while we used gills), and we detected the co‐amplification at a lower rate of a second mtCOI sequence of *M. edulis* origin in one of the two samples. Overall, although we have no histological examination of the mussels and will inevitably need to conduct further analyses, our results support the hypothesis that a cancer of *trossulus* origin may infect at a low‐prevalence mussel populations on a worldwide scale: *M. trossulus* in northwest and northeast America, and *M. edulis* in Europe. If this proves true, this infectious cancer would have accumulated mtDNA mutations, suggesting the transmissible cancer has evolved since its emergence. Nonetheless, a similar puzzle as the one initially identified with our first‐thought hybrid origin hypothesis remains as follows: How did this parasitic *M. trossulus* cancer reached Europe, far away from *M. trossulus* native distribution range, while infecting so few mussels? Although prevalence in British Columbia was also low (Metzger et al., 2016), we may nonetheless have missed less infected individuals despite our close inspection and have underestimated the prevalence in the populations we analyzed. For instance, three of the five weird genotypes missed the *M. trossulus* mtCOI co‐amplification probably because the initial proportion of cancerous cells may have been below the limit of detection. Alternatively, another unidentified species could have served as a vector, or the genome of the cancerous parasite evolved during a long period and has lost many useless genomic regions. The lack of a *M. trossulus* mtCOI sequence in three mussels suspected infected by the *M. trossulus* cancer based on nuclear SNPs could also be explained whether malignant cells have acquired mitochondria from its *M. edulis* hosts. Mitochondrial capture from the host into the cancerous lineages has indeed been described in canine transmissible venereal tumors (Rebbeck, Leroi, & Burt, 2011; Strakova et al., 2016). More investigation would however be needed to confirm this hypothesis, starting with the confirmation that neoplasia is found in French mussels (Benabdelmouna & Ledu, 2016). In any case, if transmissible cancers are widespread and unrelated to new anthropogenic modifications of the environment, we expect this newly discovered kind of parasite to have evolved for long periods before their extinction or the one of their host. For instance, in dogs, the infectious cancer lineage has been estimated to originate approximately 10,000 years ago (Rebbeck, Thomas, Breen, Leroi, & Burt, 2009), allowing the evolution of cancerous lineages. Studying the prolonged evolution of transmissible cancer genomes is likely to reveal interesting features—for example, distinguishing the core from the dispensable genomes of cancerous lineages—that may also provide insights about shorter evolutionary trajectories followed by standard single‐host cancers.

## 4 Conclusions

Metzger et al. (2016)'s publication suggests transmissible cancers could be widespread in invertebrates, and therefore genetic chimerism may be more frequent than thought. We believe population genetics not only needs to integrate this new element in the interpretation of molecular data but can also contribute to the fantastic forthcoming quest to the identification, description, investigation, and monitoring of this newly discovered kind of parasites that emerge from its host genome. We can first start by reevaluating some inexplicable patterns in available data. As population genetics is increasingly moving toward NGS‐based analyses, we will also need to develop bioinformatics routines dedicated to identify genetic chimerism in NGS data. Such methods could rely on the allelic read‐count distribution as performed to detect polyploidy with NGS data (Ament‐Velásquez et al., 2016). Indeed, we recommend routine inspection of allelic read‐count distributions to be included in the best practice of population genomics analysis.

## Data Archiving Statement

The nucleotide sequence data reported in this study have been deposited in the GenBank database under accession numbers KX925569 and KX925570.
